# From clicks to contributions: how environmental identity and impression management shape university students’ organizational citizenship behavior for the environment

**DOI:** 10.3389/fpsyg.2026.1823830

**Published:** 2026-05-12

**Authors:** Yinghe Wang, Huaming Wu, Yanbo Wang

**Affiliations:** 1 School of Art, Anhui Wenda University of Information Engineering, Hefei, China; 2 Faculty of Social Science and Humanities, The National University of Malaysia, Bangi, Malaysia; 3 School of Business Administration, Anhui University of Finance and Economics, Bengbu, China

**Keywords:** environmental identity, impression management motivation, organizational citizenship behavior for the environment, social identity theory, social media environmental engagement

## Abstract

**Introduction:**

With the growing prevalence of social media, college students’ pro-environmental participation has become increasingly important for promoting environmental sustainability. However, empirical research on how social media environmental engagement influences college students’ organizational citizenship behavior for the environment remains limited. Based on social identity theory and impression management theory, this study investigates the mechanism linking social media environmental engagement to organizational citizenship behavior for the environment among college students. It examines the mediating role of Environmental Identity and the moderating role of impression management motivation.

**Methods:**

Data were collected from 360 valid responses through a three-wave longitudinal survey. Hypotheses were tested using hierarchical regression analysis and the Bootstrap method.

**Results:**

Results show that social media environmental engagement positively predicts environmental identity among college students. In turn, environmental identity transmits the effect of social media environmental engagement onto organizational citizenship behavior for the environment. Impression Management Motivation negatively moderates the relationship between environmental identity and organizational citizenship behavior for the environment. It also weakens the mediating effect of environmental identity in the link between social media environmental engagement and organizational citizenship behavior for the environment.

**Discussion:**

These findings provide theoretical guidance for universities and social organizations to promote substantive pro-environmental behavior among college students via social media as part of their educational mission. They help facilitate the transformation from virtual engagement to real environmental action.

## 1 Introduction

Social media is reshaping young people’s engagement with environmental issues, with university students being particularly active in online environmental discussions (Pew Research Center, 2021) this digital enthusiasm often fails to translate into substantive offline actions, with studies showing a gap between online attention and real-world participation (Boulianne et al., 2020; Kristofferson et al., 2014). University students are in a critical stage of identity exploration and formation, often called emerging adulthood. For them, this gap is not only a behavioral puzzle but also an educational challenge and opportunity. This disconnect between the virtual and real world’s raises a core question: Under what conditions does social media environmental engagement (ESME) foster genuine organizational behavior, and when does it merely become “performative environmentalism”? Answering this question holds significant theoretical and practical value for advancing United Nations Sustainable Development Goal 4.7 and driving the green transition in higher education (United Nations Educational, 2020).

Existing research has three key limitations in explaining the gap between virtual participation and real-world action. First, the Theory of Planned Behavior (Ajzen, 1991) and the Value-Belief-Norm model (Stren, 2000) focus on attitudes, norms, and behavioral intentions. Yet they struggle to explain why individuals with similar attitudes behave drastically differently across contexts (Stren, 2000). Recent studies have explored social media’s impact on environmental attitudes (Hamid et al., 2017). However, they overlook a critical point. Consistent cross-context behavior does not stem from superficial cognitive change. Instead, it arises from deep self-concept restructuring—what we call Environmental Identity (EI) (Van der Werff et al., 2013; Whitmarsh and O'Neill, 2010). Second, existing literature generally assumes that social media universally promotes pro-environmental behavior. It ignores key boundary conditions. In highly visual, socially feedback-driven digital environments, individuals’ environmental expressions may stem more from impression management motivation (IMM) than genuine environmental commitment. This “greenwashing” phenomenon at the individual level is becoming more prominent (Delmas and Burbano, 2011; Torelli et al., 2020; Testa et al., 2018). However, how impression management interferes with identity’s behavioral effects lacks systematic theoretical explanation and empirical testing. Third, most existing research focuses on individual-level daily pro-environmental behaviors, such as waste sorting and energy conservation. It pays insufficient attention to collective action in organizational settings, particularly within the developmental context of higher education (Ones and Dilchert, 2012). Campus sustainability relies on Organizational Citizenship Behavior for the Environment (OCBE) among students—spontaneous contributions beyond formal responsibilities (Organ, 1988) that represent a meaningful outcome of education for sustainable development. However, university students are in a critical stage of identity formation (emerging adulthood), yet how social media engagement influences their developmentally significant OCBE remains an under-explored research gap (Raineri and Paillé, 2016). In addition to the aforementioned theoretical gaps, previous studies also have limitations in terms of methods and contexts: In terms of methods, most existing studies have adopted cross-sectional designs and lack longitudinal evidence to test the mediating role of EI; In terms of context, the existing literature is mainly based on Western samples, while non-Western contexts (such as China, which has a unique social media ecosystem) have not been fully explored.

Based on social identity theory (SIT), this study developed an integrative analytical framework and proposed that EI serves as the key mediating mechanism linking ESME and offline OCBE. Unlike surface-level cognitions such as attitudes, EI is a relatively stable self-definition. It drives consistent behavioral expression across contexts, even in the absence of external incentives (Fielding et al., 2008). Social media offers a unique accelerative mechanism for individuals to construct the EI of “being an environmentalist”. It enables continuous exposure to the normative systems and cultural symbols of environmental communities (Turner et al., 1987). When EI is internalized, individuals not only fulfill basic pro-environmental tasks, but also tend to take initiative in OCBE. Such spontaneous contributions beyond formal responsibilities are the core driving force for the sustainable development of campus environmental organizations (Boiral and Paillé, 2012). Existing studies have confirmed that EI predicts individual pro-environmental behavior far better than attitudes and norms do. Moreover, group identity formed in virtual settings exerts a behavioral driving effect similar to that of offline group identity (Van der Werff et al., 2014).

SIT believes that not all forms of group participation can give rise to genuine identity. When individuals participate for strategic considerations, what they exhibit is more likely to be a performative identity rather than a true reconstruction of their self-concept (Tajfel and Turner, 1986). Based on this view, this study further introduces IMM as a key boundary condition into the model. Social media is a highly open environment influenced by social feedback (Goffman, 2002; Kernis and Goldman, 2006). When an individual’s environmental-related expressions are dominated by IMM, their participation behavior is essentially aimed at shaping a “green image” to gain social recognition, rather than stemming from genuine identification with the environmental protection group (Leary and Kowalski, 1990). This performative identity may enhance the explicit expression of EI, but it cannot promote the in-depth internalization of identity, thus lacking the motivation to maintain behavioral consistency in different situations (Kernis and Goldman, 2006). Based on this, this study proposes the hypothesis that IMM plays a negative regulatory role between EI and OCBE. For individuals with low IMM, they can form genuine EI and effectively transform it into contributing behaviors to the organization. For individuals with high IMM, although their explicit EI levels are similar, due to the lack of deep internalization, their actual behavioral performance will be significantly weakened. Based on the above analysis, this study constructed a moderated mediation model.

The present study provides three major theoretical implications. First, it identifies EI as the core transformative mechanism connecting virtual environmental engagement and real-world pro-environmental action. This goes beyond the explanatory limits of the traditional attitude-behavior framework, and proves that cross-context behavioral transfer relies on the deep restructuring of individuals’ self-concept (Geiger et al., 2017; Ballard et al., 2017). Second, it identifies a critical boundary condition within the framework of SIT, distinguishing the divergent behavioral driving effects of genuine identity and performative identity. Our findings uncover the dual-edged influence of social media within environmental mobilization, thereby providing a systematic theoretical explanation for phenomena such as performative environmentalism and slacktivism (Kristofferson et al., 2014; Casaló et al., 2020). Third, this study incorporates organizational citizenship behavior into the investigation of pro-environmental conduct within the higher education setting. By focusing on university students’ OCBE, this study expands the research paradigm from an individual-behavior focus to an educational-outcome focus, validating that engagement with social media can affect a core indicator of education for sustainable development. At the practical level, the findings provide universities with evidence to cultivate students’ genuine environmental commitment and design educational interventions that promote authentic identity formation.

## 2 Theory and hypotheses

### 2.1 SIT and EI

SIT suggests that individuals’ self-concept is jointly shaped by two interrelated components: personal identity and social identity. Social identity is derived from individuals’ emotional and evaluative attachment to a specific social group, through which they derive a sense of belonging and commit to the values endorsed by that group (Tajfel and Turner, 1986; Tajfel and Turner, 1979). When a particular social identity becomes salient, individuals are motivated to align their attitudes and behaviors with group norms, as this helps maintain group distinctiveness and enhances their self-esteem. As an extension of social identity theory, self-categorization theory further clarifies how individuals define themselves within social contexts. Specifically, people classify themselves into relevant social groups through cognitive categorization. They then internalize the typical traits and norms of these groups via identification processes and evaluate their group relative to others through social comparison (Turner et al., 1987). These interlocking cognitive mechanisms interact dynamically and reinforce each other, which in turn promotes the development of a consistent and stable social identity.

The essential difference between identity and attitude lies in the self-defining attribute of identity: identity answers “who I am” rather than “what I believe”. Therefore, identity has a more powerful guiding effect on individual behavior (Van der Werff et al., 2013). Identity-rooted behaviors stem from the need for self-actualization and exhibit cross-context stability and spontaneity (Whitmarsh and O'Neill, 2010; Fielding et al., 2008). True identity requires individuals to maintain consistency among self-awareness, external behavior and internal experience, and this consistency is the key mechanism by which identity drives continuous behavior (Kernis and Goldman, 2006). Recent studies have further revealed that personal identity has a more significant impact on consumption behavior, while social identity has a stronger driving effect on activist behavior (Mano, 2024). From an educational perspective, these development characteristics hold significant implications. For college students, the period from adolescence to the initial emergence of adulthood (18–25 years old) is a crucial stage for identity exploration and formation (Erikson, 1968; Arnett, 2000). This group shows a stronger tendency to pay attention to environmental issues and is more inclined to construct their self-identity by participating in social movements (Ojala, 2012; Earl et al., 2017). At the same time, they highly rely on digital media for social interaction, which makes the online environment an important field for the formation of their EI.

### 2.2 ESME and EI

As a networked public space, social media has distinct technical features—persistence, replicability, searchability and an invisible audience. These characteristics profoundly shape an individual’s self-presentation and identity construction (Boyd, 2010). Its algorithmic mechanism and social network structure further give rise to the echo chamber effect, enhancing individuals’ perception of group boundaries (Pariser, 2011).

ESME fosters the formation of EI through three mechanisms. First, it strengthens the perception of group boundaries. When university students pay sustained attention to environmental issues, algorithmic recommendation mechanisms skew their social networks toward environmental communities. This reinforces the social categorization between “environmentalists” and “non-environmentalists” (Boulianne, 2019). Recent research finds that young female environmental activists construct eccentric identities through online communication channels. Hashtags related to climate action also create clear group boundaries in digital spaces (Huang et al., 2025). Second, it facilitates the process of identity self-verification. Drawing on self-determination theory, people deduce their self-identity through observing their own behavioral patterns (Bem, 1972). Environmental engagement on social media constitutes an observable public commitment. This repeated identity assertion produces a self-persuasion effect (Van der Werff et al., 2014) and further strengthens EI when peer approval is obtained (Boulianne et al., 2020; Valkenburg et al., 2006). Third, it facilitates upward social comparison. Social media allows university students to observe and compare the pro-environmental behaviors of reference groups. Such upward comparison motivates individuals to strengthen their environmental commitments, so as to maintain a positive group member identity (Festinger, 1954). Notably, virtual group identification and offline group identification are equivalent in terms of psychological mechanisms and behavioral consequences (Postmes et al., 1998). A meta-analytic review conducted by Vesely and Klöckner (2020) demonstrates that identity’s ability to predict pro-environmental behavior remains strong even after accounting for social desirability bias—evidence that the behavioral mechanism driven by identity is inherently authentic. Recent research points out that leveraging social identity is one of the effective strategies to promote pro-environmental behavior (Hrabetz et al., 2024). In light of the preceding discussion, the present research puts forward the hypotheses outlined below:

*H1*: ESME contributes positively to university students’ EI.

### 2.3 EI and university students’ OCBE

OCBE was introduced to the field of organizational research by Han et al. (2019), Boiral and Paillé (2012) further defined it as pro-environmental behavior that individuals initiate voluntarily, perform beyond formal job requirements, and advance an organization’s environmental goals. This definition emphasizes three core characteristics: spontaneity, extra-role performance, and environmental orientation. In the higher education context, Han et al. (2025a, 2025b) extended the OCBE concept to university students. They defined it as voluntary pro-environmental practices that university students take initiative to implement on campus, driven by personal values rather than institutional mandates. Specific behaviors include turning off lights voluntarily when leaving classrooms, rejecting disposable tableware, promoting environmental concepts to peers, and taking initiative to participate in environmental activities. Recent research has shown that students’ identification with their university and their environmental attitudes exert a dual mediating effect on the formation of their environmental citizenship behavior (Huang et al., 2025). University environmental organizations are typically student-managed voluntary associations. They lack formal authoritative structures and material incentive mechanisms, and their daily operation relies heavily on the spontaneous contributions of members. According to SIT, when EI becomes a core component of a person’s self-understanding, the individual develops a strong motivation for identity consistency. They tend to exhibit identity-consistent behavior across different contexts to maintain self-coherence and consistency (Stryker and Burke, 2000). For university students with a strong EI, environmental organizations are not only platforms for participating in environmental activities, but also important settings for strengthening their EI. The performance outcomes of the organization are closely associated with individuals’ self-esteem. As a result, they are more inclined to go beyond minimum requirements and take initiative to contribute to the organization (Van Dick et al., 2006).

Theoretically, EI drives university students’ OCBE through four mechanisms. First, role expansion. EI prompts individuals to expand their role perception of being an environmental organization member from completing tasks to contributing all possible efforts to the environmental cause (Ashforth, 2000; Van der Werff et al., 2014). Second, in-group favoritism. Individuals enhance their organization’s reputation and influence by engaging in OCBE, thereby indirectly boosting the perceived value of their social identity (Tajfel and Turner, 1986). Third, intrinsic motivation activation. OCBE becomes an important channel for individuals’ self-expression and self-actualization, thus demonstrating a high level of spontaneity and persistence (Ryan and Deci, 2000). Fourth, moral obligation. EI activates eccentric and altruistic values, leading individuals to develop a moral responsibility that transcends personal interests (Stren, 2000). Empirical studies to date have consistently supported a positive relationship between EI and OCBE (Raineri and Paillé, 2016; Van der Werff et al., 2014; Han et al., 2025a, 2025b). In view of the preceding arguments, this research puts forward the hypotheses presented below:

*H2*: EI among university students exerts a beneficial impact on their OCBE.

### 2.4 The mediating role of EI

Although Hypothesis 1 and Hypothesis 2 have established a sequential relationship from ESME to EI, and then from EI to OCBE, the core theoretical question remains: Why is it EI—rather than other psychological constructs (such as attitudes, norms)—that acts as the key mechanism for transforming virtual participation into real-world organizational behavior? We believe that the reason why EI uniquely suited Bridges this gap is based on the following three reasons: First, EI can achieve behavioral consistency across situations. According to the social identity theory, identity—unlike attitudes that depend on specific situations—is a stable self-definition that can drive individuals to maintain consistent behavior in different situations (Van der Werff et al., 2013; Tajfel and Turner, 1986). After an individual internalizes the label of “environmentalist” through the ESME, this self-perception transcends the limitations of the virtual context. Even in an offline organizational environment where the cost of behavior is higher and social feedback is less direct, it will prompt individuals to take actions consistent with their own identities (Kristofferson et al., 2014). Second, EI provides an intrinsic motivational force. Behaviors rooted in identity stem from the need for self-actualization rather than external incentives, and thus exhibit greater autonomy and sustainability (Ryan and Deci, 2000). Once EI is formed, students will voluntarily contribute to environmental protection organizations out of intrinsic motivation, even without external rewards or supervision. Thirdly, EI integrates multiple dimensions such as cognition, emotion and society. Unlike single cognitive variables such as attitude or self-efficacy, EI encompasses emotional attachment to nature and social identification with environmental groups. This multi-dimensionality makes it a more robust predictor of spontaneous, out-of-role behaviors (such as environmental, organizational, and civic behaviors) (Sparks and Shepherd, 1992).

Empirical research supports this mediating path. Meta-analysis studies have confirmed that the predictive effect of identity on environmental behavior exceeds that of attitude and social approval (Vesely and Klöckner, 2020). Lacasse (2016) found that EI plays a mediating role in the influence of environmental information exposure on behavior; Van der Werff et al. (2013) demonstrated that green self-identity conveys the influence of ecological values. In the educational context, the research by Han et al. (2025a, 2025b) indicates that psychological variables such as green self-efficacy play a mediating role in college students’ OCBE. Synthesizing this theoretical and empirical foundation, we propose:

*H3*: EI among university students serves as a mediating variable in the link between their ESME and OCBE.

### 2.5 The moderation effect of IMM

Although EI exerts a strong driving force on behavior, not all individuals who express an EI form a genuinely internalized one. The public nature of social media and its social feedback mechanisms create intense impression management pressure, which may lead to a divergence between performative identity and genuine identity. Impression management theory (IMT) (Goffman, 2002; Leary and Kowalski, 1990) posits that individuals selectively present information in social interactions to influence others’ perceptions of them, so as to build or maintain a desired social image. For university students, self-awareness develops rapidly in adolescence and emerging adulthood, and they are highly sensitive to others’ evaluations. Peer approval often serves as an important source of their sense of self-worth. Environmentalism is a widely socially endorsed value, and expressing environmental stances can not only win social approval, but also help shape a positive self-image (Krämer and Winter, 2008).

The technical features of social media further strengthen IMM. Every behavior of an individual may be observed by all members of their social network. Likes, comments and reposts provide an immediate measure of social approval, and individuals can also elaborate on and polish the content of their self-presentation through careful planning. Prior study has demonstrated that social media engagement patterns exert a notable impact on youth’s self-presentation strategies and their understanding of authentic self-expression (Deci and Ryan, 2013). When individuals are dominated by IMM, they tend to form a performative identity rather than a genuine inner identity. They adopt the superficial symbols of the environmental group without truly internalizing its values. Such behavior may achieve the goal of gaining social approval, but it lacks a foundation of self-definition and deep value commitment (Kernis and Goldman, 2006).

The key difference in behavioral effects between performative identity and genuine identity lies in the source of behavioral motivation. Behaviors driven by genuine identity stem from intrinsic values and autonomy (Ryan and Deci, 2000), and exhibit cross-contextual stability. In contrast, behaviors driven by performative identity derive from extrinsic motivation (Deci and Ryan, 2013), and are characterized by strategic behavioral choice (a tendency to select highly visible, low-cost behaviors), situational dependence (diminished motivation in the absence of external oversight), and fragmentation of the self-concept (Leary, 2019; Bolino, 1999). Therefore, for university students with low IMM, their EI is more likely to be a genuinely internalized one that can effectively drive OCBE. For students with high IMM, their EI is mostly a form of strategic self-presentation: while it may drive highly visible online behaviors, it hardly translates into organizational citizenship behavior that requires actual effort and is low in visibility. Existing empirical studies have also provided support for the negative moderating role of impression management in this relationship (Griskevicius et al., 2010; Bolino et al., 2008). Theoretically, IMM interacts with SIT because it determines the authenticity of identity—a condition that SIT implicitly assumes but does not explicitly theorize. SIT assumes that social identification leads to identity-consistent behavior. However, IMT reveals that when IMM is high, individuals may engage in “performative identity”: adopting group symbols without deep value internalization. Such performative identity satisfies surface identification but fails to trigger the identity-consistency mechanism that SIT posits. Hence, IMM logically moderates the EI-OCBE relationship by filtering whether an identity is genuine enough to drive behavior. In line with the above reasoning, we put forward the following hypothesis:

*H4*: University students’ IMM negatively moderates the relationship between EI and OCBE. Specifically, the higher the IMM, the weaker the positive effect of EI on OCBE.

### 2.6 The moderated mediation model

**Figure 1:**
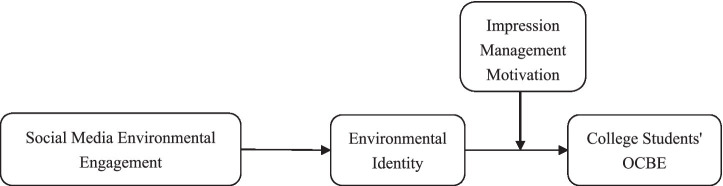
Theoretical model.

Integrating the aforementioned theoretical logic and research hypotheses, this research establishes a moderated mediation model. Drawing on the integrated framework of SIT and IMT, ESME indirectly influences university students’ OCBE through the internalization of EI, and the strength of this indirect effect is moderated by individuals’ IMM. Specifically, IMM moderates the second stage of the mediating path—that is, the effect of EI on OCBE—thus rendering the overall indirect effect conditional Figure 1.

This regulated mediation model reveals the conditional mechanism by which ESME translates into substantive organizational contribution. When IMM is low, the EI developed through ESME tends to be authentic, and this genuine identity strongly drives OCBE. When IMM is high, even if EI is expressed, it remains performative and fails to translate into OCBE. Consistent with our model, IMM moderates only the second stage of the mediation (EI—OCBE), not the first stage (ESME—EI). In the low-IMM path, ESME serves as the starting point of identity internalization, and genuine identity constitutes the psychological basis for behavioral persistence, ultimately leading to substantive organizational contribution. In contrast, for students with high IMM, ESME may enhance their superficial expression of EI, but this performative identity lacks deep value internalization and self-definition, and thus fails to stimulate cross-context identity consistency motivation. When individuals enter the organizational context—a behavioral field with low visibility and lacking immediate social feedback—performative identity cannot provide sufficient internal motivation for the OCBE that requires actual contribution, thereby leading to a significant weakening or even disappearance of indirect effects. Specifically, this study proposes that under the condition of low IMM, the indirect effect of ESME influencing OCBE through EI is significant and strong. Under the condition of high IMM, this indirect effect is significantly weakened. This difference in indirect effects indicates that identity authenticity plays a crucial role in the transformation of virtual participation into substantive actions: only when ESME gives rise to real identities can virtual participation truly be transformed into real contributions. In light of the preceding reasoning, the present study puts forward the following hypotheses:

*H5*: University students’ IMM conditions the indirect impact of ESME on OCBE through EI. Specifically, the indirect effect is significant and strong under the condition of low IMM, and attenuated under the condition of high IMM.

## 3 Method

Data were collected via the Credamo platform, a widely used professional online survey platform in China. It is accessible to corporate and academic users and features data quality control functions, having been adopted in numerous studies across the social and behavioral sciences. A three-wave longitudinal research design was used for questionnaire distribution and collection on this platform, with a two-week time lag between waves. Data collection was implemented in three waves across a five-week period, spanning September 7 to October 12, 2025. At Time 1 (T1), participants completed an initial questionnaire that assessed demographic characteristics, ESME and IMM. The questionnaire was distributed to university students through the Credamo platform. In total, 500 responses were collected, and 428 valid responses were retained after preliminary screening to form the T1 sample. To minimize attrition in this longitudinal study, appropriate incentives were provided to participants in the subsequent waves. Two weeks later, at Time 2 (T2), participants who completed the T1 survey were invited to fill out a second questionnaire measuring EI. Valid responses were obtained from 400 participants, establishing the T2 sample. After another two-week interval, at Time 3 (T3), participants in the T2 sample were asked to complete a third questionnaire assessing their OCBE. A total of 376 valid responses were collected at this stage. Responses across the three waves were matched using a unique identification code to ensure consistent participant membership. Invalid responses were excluded according to the following criteria: (a) completion time of less than 3 min; (b) failure to respond correctly to embedded attention check items; (c) logical inconsistencies in responses to reverse-worded items; (d) highly patterned or repetitive responding (e.g., zig-zag response patterns). Following rigorous data cleaning procedures, 360 matched responses were retained for the final analyses, representing a valid response rate of 72%. Attrition rates across all measurement waves were within an acceptable range.

Convenience sampling was employed in this study, with university student participants recruited from various types of higher education institutions across multiple regions in China. This sampling method was chosen for the considerations of feasibility and exploratory research. The demographic profile of the final sample is presented below: 75.2% female participants; an even distribution of undergraduate students across freshmen to senior years, with postgraduate students accounting for 24.25%; 4.9% of participants majoring in environment-related disciplines and 95.1% in non-environment-related disciplines; with 68.4% of respondents reporting more than 3 h of daily social media use.

### 3.1 Variable measurement

All variables in this study were measured using a 5-point Likert scale ranging from 1 (strongly disagree) to 5 (strongly agree). The scale adaptation followed the standard cross-cultural translation procedure established by Brislin (1980). All original English scales were subjected to back-translation to guarantee consistent meaning across the Chinese and English versions.

*ESME*: The ESME scale was adapted from the Adolescent Social Media Involvement Scale (Ni et al., 2020) and the Facebook Intensity Scale (Ellison et al., 2007). The original scales measure how extensively individuals incorporate social media into their everyday routines (e.g., social media forms a component of my daily life). Following a context-focused approach, the present study adapted the original scales to a pro-environmental context, shifting the measurement from general social media use to engagement with pro-environmental content. It covers four dimensions: browsing and reading, sharing opinions, discussing topics, and liking or commenting on relevant content. The adapted scale comprised 4 items, measuring university students’ engagement with environmental content on social media. Sample items included: I often browse and read environmental content on social media; I actively share environmental information or opinions on social media; I frequently discuss environmental topics with others on social media; I frequently engage with environmental content on social media by liking, commenting, or sharing relevant posts. Confirmatory factor analysis (CFA) was conducted via AMOS 24.0 to evaluate the construct validity of the revised scales. The results indicated that the factor loadings for all items fell between 0.66 and 0.80. The model fit statistics are presented below: *χ^2^*/df = 5.929, CFI = 0.981, TLI = 0.943, NFI = 0.978. The *χ^2^* value was statistically significant [*χ^2^*(2) = 11.86, *p* = 0.003]. However, given that the *χ^2^* statistic is highly sensitive to sample size (*N* = 360) and all other fit indices exceeded the recommended critical values (CFI > 0.95, TLI > 0.90, NFI > 0.95), the adapted scale was judged to have acceptable construct validity (Hu and Bentler, 1999). In this study, the scale demonstrated a Cronbach’s *α* value of 0.823.

*EI*: we adapted the EI scale from the revised EI Scale developed by Clayton et al. (2021). The original instrument assesses three key aspects: individuals’ emotional bond with the natural environment, the degree to which individuals perceive themselves as interconnected with nature, and how central environmental values are to their self-perception. Using a context-focused approach, the study adapted the original scale to the social media context, focusing on EI expressed by individuals on social media. The adapted scale included 6 items. Sample items were: I consider myself an environmental advocate or activist; My environmental commitments and values represent a core component of my self-concept and identity. In the present study, the scale exhibited satisfactory internal consistency reliability (Cronbach’s *α* = 0.800).

*IMM*: IMM was assessed according to the two-dimensional model of impression management (Leary and Kowalski, 1990) and the framework of self-presentation theory (Leary and Kowalski, 2012). The scale assesses the extent to which individuals attend to others’ perceptions when presenting an environmental image on social media, as well as their strategic behaviors aimed at shaping a positive image. The adapted scale consisted of 3 items. Sample items included: I carefully select the environmental content to post on social media to show my best side; Before expressing environmental opinions, I consider how this will influence others’ perceptions of me. This study recorded a Cronbach’s α value of 0.781 for the scale’s internal consistency.

*OCBE*: The scale measuring university students’ OCBE was adapted from the original Organizational Pro-environmental Behavior Scale (Boiral and Paillé, 2012). The original scale measures employees’ voluntary pro-environmental behaviors in organizational settings, including three dimensions: ecological participation, ecological initiative, and ecological helping. Referring to the study of Han et al. (2025a, 2025b) on Chinese university students, the present study adjusted the measurement context from organizational to campus settings to fit the student population. The adapted scale comprised 6 items, covering three dimensions: participation in environmental activities, daily pro-environmental behavior, and pro-environmental helping behavior. Representative items included the following: I take an active part in diverse environmental initiatives and projects hosted by my university or class; I voluntarily remind and encourage other students to engage in environmental protection even when this is not my duty. In the present study, the scale showed acceptable internal consistency, with a Cronbach’s *α* of 0.751.

*Control variables*: Following the approach of prior research (Carducci et al., 2021), the study controlled for several demographic variables that could potentially shape students’ pro-environmental actions. The control variables were as follows: gender (1 = male, 2 = female); academic year (1 = freshman, 2 = sophomore, 3 = junior, 4 = senior, 5 = postgraduate); major background (1 = environment-related majors, e.g., environmental science, ecology; 2 = non-environment-related majors); daily social media usage time (1 = less than 1 h, 2 = 1–3 h, 3 = 3–5 h, 4 = more than 5 h).

## 4 Results

### 4.1 Confirmatory factor analysis

We conducted confirmatory factor analysis using IBM AMOS 24.0. Four competing measurement models were tested and compared: a four-factor model (ESME, EI, IMM, and OCBE), a three-factor model, a two-factor model, and a single-factor model. The findings demonstrated that the four-factor solution provided a superior fit to the data (*χ^2^* = 246.009, df = 146, *χ^2^*/df = 1.685, CFI = 0.957, TLI = 0.950, RMSEA = 0.044, SRMR = 0.042) (Table 1). Reliability and validity were assessed using Cronbach’s α, composite reliability (CR), average variance extracted (AVE), and the heterotrait-monotrait ratio (HTMT). As shown in Table 2, Cronbach’s α values ranged from 0.751 to 0.823, and CR values ranged from 0.759 to 0.830, all of which were above the commonly accepted threshold of 0.70. Following the guidelines established by Hair et al. (2010), convergent validity was evaluated using the thresholds of AVE ≥ 0.50 and CR ≥ 0.70. Results showed that AVE values for ESME (0.551) and IMM (0.544) satisfied the 0.50 criterion, whereas those for EI (0.410) and OCBE (0.349) fell below this benchmark. Nevertheless, consistent with the decision rule outlined by Fornell and Larcker (1981), convergent validity for EI and OCBE remained acceptable because their CR values (0.806 and 0.759, respectively) were both above 0.70. The relatively low AVE values for EI and OCBE were consistent with theoretical predictions, as both constructs are conceptually multidimensional. EI may reflect individual differences in cognitive and affective identification with the environment, and OCBE encompasses various forms of spontaneous pro-environmental behaviors. For discriminant validity, the HTMT criterion was further adopted for testing. The HTMT values among all constructs varied from 0.331 to 0.805, all of which were lower than the standard critical thresholds (0.85 and 0.90), thus supporting adequate discriminant validity between the constructs.

**Table 1:** Comparison of measurement models.

Model	*χ^2^*	df	*χ^2^*/df	CFI	TLI	RMSEA	SRMR
Four-factor model (ESME, EI, IMM, and OCBE)	246.009	146	1.685	0.957	0.950	0.044	0.042
Three-factor model (ESME+EI, IMM, and OCBE)	405.084	149	2.719	0.891	0.874	0.069	0.056
Two-factor model (ESME+EI + IMM, OCBE)	625.550	151	4.143	0.797	0.770	0.094	0.074
Single-factor model (ESME+EI + IMM + OCBE)	660.986	152	4.349	0.782	0.755	0.097	0.075

*N* = 360.

**Table 2:** Reliability and validity of the scales.

Variables	α	CR	AVE	HTMT-EI	HTMT-IMM	HTMT-OCBE
1. ESME	0.823	0.830	0.551	0.714	0.331	0.707
2. EI	0.800	0.806	0.410		0.505	0.805
3. IMM	0.781	0.781	0.544			0.446
4. OCBE	0.751	0.759	0.349			

*N* = 360; ESME, Social media environmental engagement; EI, Environmental identity; IMM, Impression management motivation; OCBE, Organizational citizenship behavior for the environment.

### 4.2 Common method bias

Harman’s one-factor test was adopted to examine common method bias, with exploratory factor analysis conducted on all key study variables. With eigenvalues greater than 1 and no rotation performed, the first principal component accounted for 35.423% of the total variance, a value below the 40% threshold. This result suggested that common method bias had no significant impact on the empirical findings. In addition, we established a baseline confirmatory factor analysis model (M1) and a comparative model (M2) that included a common method factor. The key fit indices of M1 and M2 were compared, with the results showing that Δ*χ^2^*/df = 0.256, ΔCFI = 0.02, ΔTLI = 0.019, and ΔRMSEA = 0.009. The changes in CFI and TLI were both less than 0.1, and the change in RMSEA did not exceed 0.05. These results demonstrated that adding the common method factor did not significantly enhance model fit, indicating no substantial common method bias in the measures. Overall, no meaningful common method bias was identified in this study, and its impact on the final results was negligible.

### 4.3 Correlation analysis

As presented in Table 3, ESME was positively and significantly associated with EI (*r* = 0.591, *p* < 0.01) and IMM (*r* = 0.264, *p* < 0.01). Additionally, ESME was positively and significantly linked to OCBE among university students (*r* = 0.565, *p* < 0.01). Moreover, EI was significantly and positively correlated with university students’ OCBE (*r* = 0.697, *p* < 0.01), and IMM exhibited a positive and significant correlation with OCBE among university students (*r* = 0.334, *p* < 0.01). The above results provided preliminary support for the research hypotheses of this study.

**Table 3:** Means, standard deviations, and correlation coefficients of the variables (*N* = 360).

Variables	M	SD	1	2	3	4	5	6	7
1.gender	1.76	0.425	1						
2.grade	3.48	1.208	−0.006	1					
3.major	1.96	0.194	−0.044	−0.015	1				
4.time	2.97	0.800	0.054	−0.107*	0.065	1			
5. ESME	3.104	0.689	−0.092	−0.089	−0.105*	0.030	1		
6. EI	3.662	0.504	−0.075	−0.004	−0.054	−0.039	0.591**	1	
7. IMM	3.728	0.674	−0.108*	−0.009	−0.053	0.063	0.264**	0.369**	1
8. OCBE	3.731	0.422	−0.035	0.014	−0.038	−0.019	0.565**	0.697**	0.334**

*p < 0.05, **p < 0.01.

### 4.4 Hypothesis testing

This study employed hierarchical regression analysis to test the research hypotheses, as it is particularly suitable for model involving mediation and moderation with longitudinal data. The results are shown in Table 4. This method can gradually test the incremental explanatory power of new variables (such as mediating variables and moderating variables) on the dependent variable on the basis of controlling confounding variables. It is suitable for evaluating the changes in the model’s explanatory power after multiple predictor variables are introduced in sequence, and thus is used for hypothesis verification. To prevent multicollinearity from unduly influencing the regression results, the variance inflation factor (VIF) was computed for all key variables. The results indicated that the highest VIF value across the main research variables was 1.708, which was far below the critical value of 5. This indicated that multicollinearity had no significant effect on the regression equations.

**Table 4:** Results of hierarchical regression analysis.

Variables	EI			OCBE		
	Model 1	Model 2	Model 3	Model 4	Model 5	Model 6
Gender	−0.090	−0.022	−0.035	0.020	0.023	0.017
Grade	−0.004	0.020	0.004	0.023	0.006	0.008
Major	−0.145	0.024	−0.083	0.054	0.008	0.008
Time	−0.021	−0.010	−0.007	0.001	0.002	0.007
ESME		0.435^**^		0.352^**^		
EI					0.556^**^	0.556^**^
IMM					0.044	0.023
EI*IMM						−0.119^*^
*R^2^*	0.010	0.352	0.003	0.324	0.491	0.505
F	0.895	38.461^**^	0.278	33.929^**^	56.774^**^	51.332^**^

*p < 0.05, **p < 0.01.

Model 1 and Model 3 are baseline models containing only control variables for EI and OCBE, respectively. SPSS was used to conduct the hypothesis tests, and the regression results are reported in Table 4. Hypothesis 1 proposed that ESME is positively associated with EI. As indicated in Model 2 of Table 4, ESME had a significantly positive effect on EI (*b* = 0.435, *p* < 0.01), thereby providing support for Hypothesis 1. Hypothesis 2 posited that EI has a significant positive effect on university students’ OCBE. As shown in Model 5 and Model 6 of Table 4, after controlling for other variables, EI exerted a significantly positive effect on OCBE in both models (Model 5: *b* = 0.556, *p* < 0.01; Model 6: *b* = 0.556, *p* < 0.01), supporting H2.

Further analyses revealed that ESME was positively and significantly related to OCBE (Model 4: *b* = 0.352, *p* < 0.01). After the inclusion of EI (Model 5), the effect of ESME decreased to 0.023 (*p* > 0.05), indicating that EI serves as a partial mediator in the link between ESME and OCBE. Bootstrap testing results (see Table 5) indicated that the indirect effect was 0.204, with a 95% confidence interval [0.163, 0.252] that excluded zero. The direct effect was 0.143, with a 95% CI [0.092, 0.194]. The mediating effect constituted 58.7% of the total effect, further confirming that EI exerts a significant partial mediating role between ESME and OCBE. H3 was thus supported.

**Table 5:** Total, direct, and indirect (mediating) effects.

	Effect size	Standard error	95% CI lower	95% CI upper	Relative effect size
Total effect	0.346	0.035	0.277	0.415	
Direct effect	0.143	0.026	0.092	0.194	41.3%
Indirect effect	0.204	0.024	0.163	0.252	58.7%

**Figure 2:**
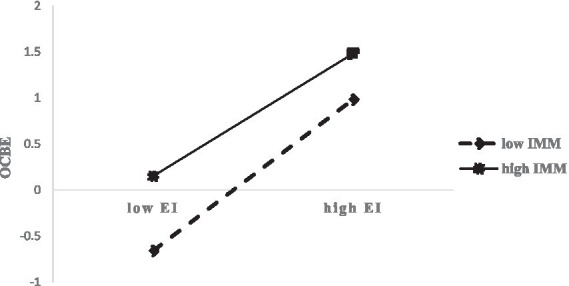
Moderating effect of IMM.

Model 6 demonstrated that the interaction term between EI and IMM was significantly negatively associated with OCBE (*b* = −0.119, *p* < 0.05). Simple slope analysis (see Figure 2) indicated that the positive effect of EI on OCBE was stronger when IMM was at a low level, and this effect weakened when IMM was at a high level. This finding indicated that IMM negatively moderates the association between EI and OCBE, suggesting that the moderating effect operates at the second stage of the mediating pathway. Hypothesis 4 was thus supported.

The moderated mediation model was examined via the PROCESS macro (Model 14) in SPSS, with the results as follows: when IMM was at a low level (IM = 3.080), the indirect effect of ESME on university students’ OCBE via EI was 0.229 [95% CI = (0.183, 0.283)], which did not include zero; when IMM was at a moderate level (IM = 3.752), the value of the indirect effect was 0.204 [95% CI = (0.163, 0.252)], which did not include zero; when IMM was at a high level (IM = 4.425), the indirect effect had a value of 0.179 [95% confidence interval (95% CI) = (0.132, 0.229)], with this interval excluding zero. The moderated mediation index was −0.037 [95% CI = (−0.073, −0.008)], and this interval also did not contain zero. These results indicated that the indirect effect of ESME on OCBE through EI gradually weakens as the level of IMM increases, meaning IMM negatively moderates the entire mediating process. H5 was supported.

## 5 Discussion

### 5.1 Discussion of the findings

This study explored the mechanism and boundary conditions by which ESME promotes OCBE in college students. The research results reveal the psychological mechanism, boundary conditions and development significance of this transformation process. Consistent with H1 and H2, ESME significantly predicts EI, and EI in turn drives OCBE. These findings confirm that online environmental protection participation can promote deep identity internalization rather than remaining at the superficial level (Turner et al., 1987; Tajfel and Turner, 1986); and this internalized identity—once formed—can stimulate spontaneous and out-of-role contributions across situations (Han et al., 2025a, 2025b; Sparks and Shepherd, 1992). It is worth noting that this study challenges the traditional doubt that virtual participation lacks genuine commitment. It confirms that the identity formation mechanism observed in offline groups is also effective in algorithm-driven online environments (Halupka, 2014). From a developmental perspective, this is particularly important for college students in the early stage of adulthood—a crucial period when identity exploration and commitment shape lifelong behavioral patterns (Erikson, 1968; Arnett, 2000). Social media, as the main social arena for this age group, thus plays a developmental role: it offers a low-risk “mental gym” where students can rehearse in advance before integrating environmental values into their self-concept.

The mediating role (H3) of EI responds to a core debate in digital activism research: under what conditions can virtual participation be transformed into real action? This study found that ESME can promote OCBE only when it first nurtures EI. When EI is formed, students’ online participation goes beyond information consumption. It also reshapes students’ self-concept. This mechanism explains the paradox of lazy activism (Kristofferson et al., 2014): The reason why symbolic online support fails to generate offline action is precisely because it fails to activate the deep internalization of identity. In contrast, when ESME successfully builds EI, it generates an intrinsic motivation that transcends situational boundaries, making students willing to contribute to environmental protection organizations even without external incentives (Ryan and Deci, 2000). From an educational perspective, this discovery emphasizes that universities should not merely focus on promoting online environmental protection activities, but also design experiences that facilitate identity integration—helping students connect virtual participation with their developing self-awareness. In addition, the samples of this study have specific generational characteristics. All the participants were college students, belonging to the Gen Z group, and grew up in an era when climate change information was highly accessible. This explains why the average values of EI and OCBE in this study were at a medium to high level. This generation was exposed to environmental issues earlier. It was also more inclined to incorporate the identity of “environmentalist” into their self-concept. Therefore, the promoting effect of ESME on EI may be particularly significant during the early adult manifestation period when identity exploration is active.

Finally, the moderating effects of IMM (H4, H5) reveal a key boundary condition: the transformation potential of ESME depends on identity authenticity. When IMM is low, the EI truly internalized through ESME will strongly drive OCBE. However, when the IMM is high, students may develop a performative identity—adopting the external symbols of environmentalism but lacking a deep value commitment (Kernis and Goldman, 2006; Krämer and Winter, 2008). From the perspective of IMT, the EI formed by individuals with high IMM is closer to a “performative identity.” That is, they adopt the external symbols of the environmental protection group but lack deep value internalization. This is in line with the logic of “slacktivism”: highly visible symbolic support may instead weaken substantive action. This kind of performative identity can maintain highly visible online expression, but it cannot inspire organizational contributions that require continuous effort and have low visibility. From the perspective of educational psychology, this reveals a key developmental challenge during the nascent stage of adulthood. A high IMM may lead to premature identity closure (premature commitment without true exploration) or identity fragmentation (incoherent switching of self-concepts between different situations) (Arnett, 2000). Therefore, the educational task lies in helping students move from performative exploration to authentic integration.

### 5.2 Theoretical contributions

This study presents three theoretical insights of significant importance in the field of educational psychology. First, this study extends the research on the formation mechanism of EI to the context of Chinese social media and the early emergence stage of its development. Previous studies have confirmed that EI can predict pro-environmental behavior (Fielding et al., 2008). However, less attention has been paid to how algorithm-driven ESME promotes the internalization of EI. This is especially true for Chinese college students. They are in a critical period of identity exploration and highly dependent on digital platforms for social interaction. This study demonstrates that ESME effectively promotes the internalization of EI through mechanisms such as algorithm-driven social classification, public commitment, and upward social comparison (Schwegler et al., 2024). This discovery reveals the unique function of social media as a developmental context in early adulthood and provides empirical evidence for the applicability of SIT in non-Western cultural contexts (Boulianne, 2019).

Second, this study does not simply claim that EI is a universal mechanism. Instead, within the integrated framework of SIT and IMT, it clearly defines it as an intermediary mechanism that connects virtual participation with substantive actions. A large number of studies in environmental psychology have discussed identity-based behavior. However, few have systematically examined how EI plays a mediating role between ESME and OCBE. Even fewer have considered the boundary conditions imposed by IMM. This study found that EI was a significant mediating variable, explaining why some students transformed their online environmental participation into actual organizational contributions, while others did not (Kristofferson et al., 2014). This discovery indicates that identity-based learning is more likely to achieve cross-context transfer than attitude-based learning. This provides a theoretical basis for designing educational interventions. Such interventions should be centered on self-concept rather than merely focusing on knowledge imparting. Furthermore, this discovery demonstrates how IMT logically extends SIT by specifying a key boundary condition. That condition is this: identity must be authentic—not merely expressed—to activate the identity consistency mechanism that SIT assumes.

Third, this study reveals the negative moderating effect of IMM on the relationship between EI and OCBE, thereby distinguishing the behavioral consequences of real identity and performative identity in the context of social media. Previous studies on IMM mainly focused on its direct effect on self-presentation, with less attention paid to how it moderates the relationship between identity and behavior. This study indicates that when IMM is relatively high, the positive effect of EI on OCBE significantly weakens. This suggests that performative identity cannot drive substantive actions. Performative identity means merely adopting the external symbols of environmentalism without deep internalization of values. From the perspective of educational psychology, this discovery clarifies under what conditions identity has an autonomous incentive effect, thereby expanding the theory of self-determination. Meanwhile, this study also indicates that assessing the degree of students’ identity integration can more effectively predict long-term behavioral outcomes. This is compared with measuring surface attitudes or explicit behaviors. Identity integration refers to the extent to which values are internalized into self-concepts (Hayes, 2015).

### 5.3 Practical implications

First, create identity-oriented learning experiences. Educators should not merely view social media as an informational tool but integrate it into curricula as a practical arena for identity exploration. Reflective writing assignments can guide students to connect online participation with personal values; structured peer discussions can reinforce the social validation component in identity formation. These approaches transform social media from a distraction into a developmental resource.

Second, establish a developmental pathway from virtual actions to real-world behaviors. Based on this study’s stepwise engagement model, educators can design progressive activities embedded within curricula: beginning with low-barrier online behaviors (such as organizing and disseminating environmentally relevant content), gradually transitioning to structured offline projects (such as campus sustainability assessments), and ultimately extending to leadership roles (such as organizing environmental campaigns). This scaffolding approach respects the identity formation patterns of early adulthood and provides students with stepwise opportunities to practice environmental commitments across diverse contexts.

Third, expand evaluation frameworks to measure identity integration levels. Traditional metrics often focus on knowledge acquisition or behavioral frequency. Findings indicate that identity integration—the degree to which environmental values become internalized as part of students’ self-concept—more effectively predicts long-term engagement. Educators can employ reflective portfolios or identity narratives for assessment, shifting focus from what students “know” and “do” to what kind of people they “become.” This paradigm shift fosters authentic, enduring intrinsic commitment rather than superficial compliance.

### 5.4 Limitations and future research directions

This study has several limitations, but it also points out a feasible direction for future research, especially from the perspective of educational psychology. First, although the three-wave time-delay design helps to strengthen causal inference, it still cannot fully establish causal relationships. Students with a relatively high EI may selectively be exposed to environmental protection content, thereby generating a two-way impact. This issue will reduce the credibility of intervention design: in the absence of clear causal evidence, educators find it difficult to develop relevant programs that take ESME as the entry point to enhance EI and OCBE with confidence. Future research can adopt experimental designs (for example, randomly assigning classes to social media-based courses) and conduct follow-up studies covering the entire academic year to reveal the development process of early adulthood identity.

Second, although EI is a key mediator, it is merely one of many psychological pathways. Future research can examine parallel mechanisms, such as self-efficacy (Han et al., 2025a, 2025b) and moral norms. For educational psychology, the more crucial next step is to explore its development mechanism—how EI changes at different educational stages. Are there any differences between freshmen and seniors in the way they influence EI on social media? Is there a difference in stability between EI formed through courses and EI formed through extracurricular activities? Cross-grade horizontal comparison or vertical tracking can provide a basis for phased and age-appropriate intervention.

Third, this study only examined IMM as the moderating variable. Future research should explore multiple regulatory factors, including individual differences, organizational factors and cultural contexts. From an educational perspective, it is equally important to examine the moderating variables at the teaching level: how curriculum design and teaching strategies affect the ESME—EI—OCBE chain of action. Will reflective writing enhance the role of ESME in EI? Will cooperative projects enhance the connection between EI and OCBE? Classroom experiments can examine the moderating effect of specific educational practices on these relationships.

Fourth, all the data are from self-reports. Although the common method bias is not serious, the AVE values of EI and OCBE are relatively low, suggesting that such topics with high social approval may have a common source bias. Future research can incorporate multi-source data (such as peer assessment, behavioral tracking, etc.) to cross-validate the results.

Fifth, the use of convenient sampling limits the generalizability of the research conclusion. Convenience sampling is a non-probability sampling method, which may have selective bias and limit the extent to which the conclusion can be generalized to all college students in China. Future research should adopt probability sampling methods (such as stratified random sampling) to enhance external validity.

## Data Availability

The raw data supporting the conclusions of this article will be made available by the authors, without undue reservation.
